# Comparison of Serum Matrix Metalloproteinase-9 Levels in Diabetic and Non-diabetic Chronic Wounds

**DOI:** 10.7759/cureus.93672

**Published:** 2025-10-01

**Authors:** Jenny Joseph, Alakhananda Chandranaath, Joel Louis, Shaikh Afzal Rubby, Vimal K Govindan, Merlin Veronika

**Affiliations:** 1 Internal Medicine, Norwalk Hospital, Norwalk, USA; 2 General Surgery, PSG Institute of Medical Sciences and Research, Coimbatore, IND; 3 Internal Medicine, PSG Institute of Medical Sciences and Research, Coimbatore, IND; 4 PSG Centre for Research and Bioethics, PSG Institute of Medical Sciences and Research, Coimbatore, IND

**Keywords:** diabetic chronic wounds, glycemic control (hba1c), matrix metalloproteinase-9 (mmp-9), non-invasive test, serum biomarker, wound healing

## Abstract

Background: Wound healing relies on a balance between extracellular matrix remodelling by matrix metalloproteinases (MMPs) and tissue inhibitors of metalloproteinases (TIMPs). Among all the MMPs, elevated MMP-9 levels are implicated in chronic wounds including diabetic ulcers, making it a potential biomarker to predict wound healing. This in turn can help determine the need for aggressive wound care management. However, most studies used invasive sampling methods like wound fluid or tissue biopsy limiting clinical implementation. Hence, this study assessed the correlation between serum levels of MMP-9 in diabetic and non-diabetic chronic wounds to explore if non-invasive methods could be used to predict healing in diabetic chronic wounds.

Objective: This study aimed to compare serum MMP-9 levels in diabetic vs. non-diabetic chronic wounds, assess correlations with glycemic control (glycated hemoglobin (HbA1c)), and determine the potential of serum MMP-9 as a predictor of healing in diabetic chronic wounds.

Methods: This was a cross-sectional study involving 40 patients (20 diabetics, 20 non-diabetics) with chronic wounds. Serum MMP-9 levels were measured from blood samples (avoiding invasive methods) using an enzyme-linked immunosorbent assay (ELISA). HbA1c levels were obtained for patients with diabetes mellitus. Statistical analysis compared means (t-test) and correlations (Pearson's).

Results: Patients with diabetes mellitus had significantly higher serum MMP-9 (mean 21.22 ng/ml) in comparison to non-diabetics (17.35 ng/ml; p=0.0023). When compared to only 5% (one out of 20) of non-diabetics, about 75% (15 out of 20) of diabetics had MMP-9 greater than 20 ng/ml. However, no correlation was found between MMP-9 and HbA1c levels (p>0.05) among diabetic patients.

Conclusions: Serum MMP-9 is elevated in diabetic chronic wounds, supporting its role as a non-invasive biomarker. Several studies have established wound fluid/tissue MMP-9 as a predictor of healing in diabetic ulcers, and this study suggests that serum MMP-9 levels could be used as a non-invasive biomarker in place of cumbersome methods. However, the lack of association of MMP-9 with HbA1c suggests glycemic control alone may not regulate MMP-9. Targeting MMP-9 inhibition (e.g., via microRNAs) could be a therapeutic strategy, warranting further research.

## Introduction

Wounds remain a demanding surgical problem leading to increased morbidity and mortality [1,2]. Thus, accurate and efficient management of wounds is of prime importance. Wound healing is a complex process that requires a balance between the accumulation of extracellular matrix (ECM) components and their remodelling by matrix metalloproteinases (MMPs) and tissue inhibitors of metalloproteinases (TIMPs) [3]. The interruption of this series of events can lead to chronic ulcers. Among MMPs, MMP-9 levels are increased in most chronic wounds and can be used as a potential biomarker [4,5]. Similar to other types of chronic ulcers, diabetic ulcers have a prolonged inflammatory phase with significant increases in pro-inflammatory cytokines, proteases, and neutrophil elastase [6]. This process becomes exaggerated and persists for a prolonged time leading to high levels of MMPs.

MMPs are a family of zinc-dependent endopeptidases. One of their major characteristics is their ability to degrade various ECM components. They are produced by several different types of cells in the skin including fibroblasts, keratinocytes, macrophages, endothelial cells, mast cells, and eosinophils. Their activity is specifically inhibited by TIMPs [7-9]. Studies on wound healing in diabetic foot ulcers have shown that serum MMP-9 was lower in healing ulcers in contrast to non-healing ulcers after a four-week therapy [10].

Lobmann et al. [11] in 2002 compared the concentration of MMP and TIMP in diabetic and non-diabetic acute wounds. The study demonstrated that the levels of MMP-2 were two- to six-fold higher and MMP-9 were 14-fold significantly higher in diabetic foot ulcers. The study also provided evidence for the reduced expression of TIMP-2 in diabetics than in non-diabetics. Among the proteases that have been reported to be increased in chronic wounds are the MMPs, specifically MMP-9, highlighting its utility as a biomarker [4,5].

Caley et al. [12] provided an elaborate description of proteases and their molecular biology and role in wounds and the estimation of MMPs. They concluded that MMPs play a crucial role in wound healing. Yet, excessive MMP expression seen in chronic wounds inhibits wound closure. Since MMPs are substrate-specific, a particular growth factor may regulate multiple different MMPs making them hard to target for therapies. Newer research delves into methods such as signal transduction, peptides, and microRNA (MiRs) to modulate MMP expression and its activity. A few inhibitory antibodies are being developed to target MMPs, such as GS-5745, which targets MMP-9 [13].

In this study, we established a link between high levels of serum MMP-9 and chronicity of wounds in diabetics and non-diabetics, by comparing and analyzing these levels between the two groups. We also checked if the levels of serum MMP-9 correlated with glycated hemoglobin (HbA1c) levels suggesting the control of diabetes.

## Materials and methods

This study was conducted at PSG Institute of Medical Sciences and Research in Coimbatore, Tamil Nadu, India, involving patients of Asian-Indian ethnicity. Ethical approval was obtained from the Institutional Human Ethics Committee of PSG Institute of Medical Sciences and Research (approval number: PSG/IHEC/2019/Appr/Exp/300). Written informed consent was obtained from all participants prior to inclusion in the study.

The study design was a cross-sectional study performed over a year (1/10/2020-5/15/2021). A total of 40 adult patients aged between 18 and 70 years with chronic ulcers (defined as wounds persisting for more than six weeks) and wound sizes ranging from 5 cm to 20 cm were enrolled. The sample size was calculated using the following parameters: (i) test family (t-test), (ii) independent sample groups, (iii) two-tailed, (iv) an effect size of 0.44 with a significance level/alpha of 0.05, and (v) an 80% power which was a total of 166 patients. However, the sample size was reduced to 40 patients due to the timing of the study during the COVID-19 pandemic and budget constraints. The study population was matched for age and gender and divided into two cohorts: 20 patients with diabetes mellitus and 20 non-diabetic patients. Exclusion criteria included the presence of rheumatoid arthritis, coronary artery disease, underlying infections like osteomyelitis and abscesses, malignancy-associated wounds, and peripheral artery disease.

HbA1c levels were obtained from all patients in the diabetic group. Blood samples were collected from all participants for the estimation of serum MMP-9 levels. Samples were analyzed using a commercially available enzyme-linked immunosorbent assay (ELISA) kit to measure MMP-9 levels. Serum MMP-9 levels were then compared between the diabetic and non-diabetic cohorts and subjected to statistical analysis to assess differences.

Serum MMP-9 levels were measured using a commercially available sandwich ELISA kit, according to the manufacturer's protocol. Serum samples, obtained by centrifuging clotted blood at 2000 rpm for 10 minutes, were stored at -80°C until analysis. Samples and standards were added in duplicate to wells pre-coated with anti-MMP-9 antibodies, followed by incubation with a biotin-labelled detection antibody and streptavidin-horseradish peroxidase. After washing, the tetramethylbenzidine substrate was added to initiate the colorimetric reaction, which was stopped with sulfuric acid. Absorbance was read at 450 nm using a microplate reader, and MMP-9 concentrations were determined by comparison to a standard curve. 

Statistical analysis

Serum levels of MMP-9 in diabetic and non-diabetic chronic wounds were analyzed for statistical significance. Continuous variables were represented as mean±standard deviation, and categorical variables were represented as frequencies and percentages. Student's t-test was used to compare the serum MMP-9 levels between the two groups. Pearson's correlation method was used to analyze the relation between HbA1c levels and serum MMP-9 levels in the diabetic group. All the statistical computations were performed using R software version 4.4.1 (R Foundation for Statistical Computing, Vienna, Austria). 

## Results

The study population was equally distributed between the two groups, namely, diabetics and non-diabetics, and was matched for age and gender. 

**Figure 1:**
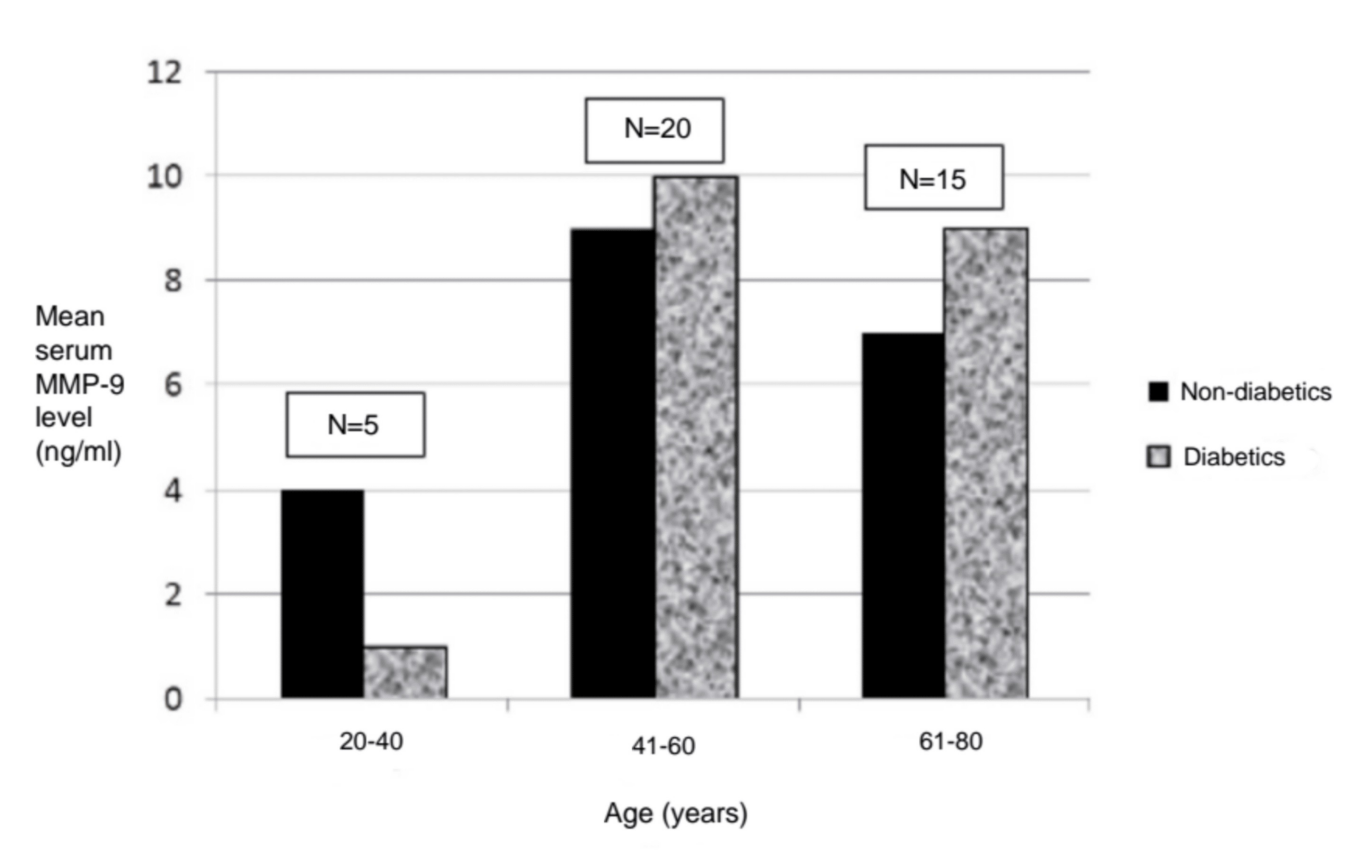
Distribution of the study population according to age group MMP-9: matrix metalloproteinase-9

Among participants in the 20-40-year age group, the mean serum MMP-9 was 4 ng/ml in non-diabetics and 1 ng/ml in diabetics. In the 41-60-year age group, the mean serum MMP-9 was 9 ng/ml in non-diabetics and 10 ng/ml in diabetics. The mean serum MMP-9 was noted to be 7 ng/ml in non-diabetics and 9 ng/ml in diabetics in the 61-80-year age group (Figure 1).

**Figure 2:**
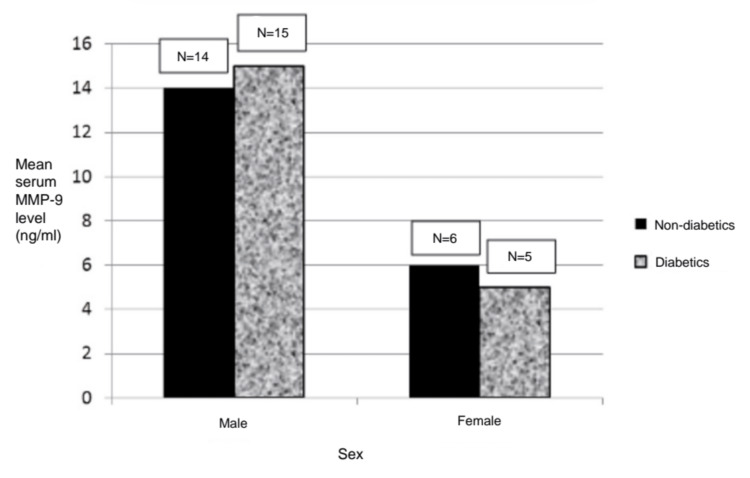
Distribution of the study population according to sex MMP-9: matrix metalloproteinase-9

The classification of the study groups by gender showed that non-diabetic males had a mean MMP-9 of 14 ng/ml, whereas diabetic males had a mean of 15 ng/ml. While non-diabetic females had a mean MMP-9 of 6 ng/ml, the mean for diabetic females was 5 ng/ml (Figure 2).

Patients with diabetes mellitus had significantly higher serum MMP-9 levels (Table 1). While comparing patients who had serum MMP-9 levels in excess of 20 ng/ml, it was significant to note that there were 75% of patients with diabetes mellitus (15 out of 20), while there were only 5% (one out of 20) non-diabetics. Additionally, 30% of diabetics (six out of 20) had serum MMP-9 levels exceeding 25 ng/ml, while there were no non-diabetics with such high levels.

**Table 1:** Distribution of the study population in both groups according to serum MMP-9 levels MMP-9: matrix metalloproteinase-9

Serum MMP-9 level (ng/ml)	Non-diabetic group (N=20)		Diabetic group (N=20)	
	Frequency	Percentage	Frequency	Percentage
<15	2	10	3	15
15-20	17	85	2	10
20-25	1	5	9	45
>25	0	0	6	30

The mean serum MMP-9 levels in the two groups, namely, non-diabetics and diabetics, were 17.35 and 21.22 ng/ml, respectively. This was found to be statistically significant with a p-value of 0.0023 (Table 2).

**Table 2:** Mean serum MMP-9 in the non-diabetic and diabetic groups Statistical tool: Student's t-test for independent groups MMP-9: matrix metalloproteinase-9

	Serum MMP-9 (ng/ml)	P-value	t-value
Non-diabetic group (N=20)	17.35	0.0023	-3.348
Diabetic group (N=20)	21.22		

The study was unable to establish a correlation between the HbA1c levels in diabetics and the serum MMP-9 levels. Nine patients with diabetes mellitus had a HbA1c in excess of 10%, of whom seven had a serum MMP-9 in excess of 20 ng/ml (Table 3). However, this had no statistical significance, and the study was unable to establish a correlation between higher HbA1c levels and serum MMP-9 values.

**Table 3:** Comparison of serum MMP-9 levels and HbA1c levels in the diabetic group (N=20) Statistical tool: Pearson's product-moment correlation. P-value=0.3801; correlation coefficient=-0.207 MMP-9: matrix metalloproteinase-9; HbA1c: glycated hemoglobin

	Serum MMP-9 levels (ng/ml)				Total
	<15	15-20	20-25	>25	
HbA1c levels (%)					
<10	1	2	4	4	11
10-15	1	1	2	2	6
15-20	0	0	3	0	3
Total	2	3	9	6	N=20

## Discussion

Muller et al. conducted a study in 2008 estimating levels of MMP-1, MMP-2, MMP-8, MMP-9, and TIMP-1 in wound fluids of diabetic foot ulcers [14]. It showed that good healing requires high levels of MMP-1, whereas MMP-8 and MMP-9 proved to be detrimental and so could be possible targets for newer topical treatments. MMP-9 is expressed in considerably higher levels in chronic wounds of diabetic patients, thus emphasizing its role in their pathogenesis [10,15].

However, most of the previous studies regarding MMPs and chronic wounds have used wound fluid [11], biopsies and smears taken from wound edges [4], and wound tissue bits as samples. Only a few studies determine plasma or serum MMPs in chronic wound patients. This study demonstrates that serum MMP-9 levels are significantly elevated in diabetic chronic wounds compared to non-diabetic counterparts (21.22 ng/ml vs. 17.35 ng/ml; p=0.0023), reinforcing MMP-9's role as a key mediator of pathological wound healing and potential as a biomarker for wound healing. Crucially, 75% of diabetic patients exceeded the serum MMP-9 threshold of 20 ng/ml versus only 5% of non-diabetics, suggesting this cut-off may hold clinical utility for identifying high-risk wounds. This aligns with prior work by Lobmann et al. [11] who documented 14-fold higher MMP-9 in diabetic foot ulcer tissue. 

Shetty et al. [16] demonstrated that higher levels of tissue MMP-9 were evident in non-healing diabetic foot ulcers when compared to healing ones. Methodologically, our serum-based approach advances the field beyond cumbersome wound fluid/tissue biopsies [4,11,14]. Muller et al. [14] and Jindatanmanusan et al. [5] previously correlated wound fluid MMP-9 with poor outcomes, but their techniques limit clinical adoption. Serum quantification offers practical advantages: standardization across facilities, integration into routine blood work, and dynamic monitoring of treatment response. Future studies should validate optimal sampling timepoints and kinetic profiles during wound progression.

Studies conducted on diabetic foot ulcers have highlighted a rising trend of MMP-9 levels in wound fluid with increasing severity of the ulcer [17]. However, we were unable to find a correlation between HbA1c values and serum MMP-9 levels of diabetic patients in this study. The absence of correlation between serum MMP-9 and HbA1c (p>0.05) or diabetes duration presents a paradigm-shifting insight, although these results could be skewed by the small sample size. While hyperglycemia is traditionally implicated in impaired wound healing [6], our data imply that MMP-9 elevation operates through glycemia-independent pathways. Potential mechanisms include chronic inflammation perpetuated by senescent cells [12], dysregulated neutrophil extracellular trap (NET) formation, or epigenetic modifications altering MMP-9 transcription. This decoupling from glycemic control underscores why intensive glucose management alone often fails to resolve diabetic ulcers and highlights MMP-9 as a standalone therapeutic target.

Wang et al. published a study highlighting that microRNA-129 and microRNA-335 promote diabetic wound healing by inhibiting sp1-mediated MMP-9 expression [17]. It has been pointed out that wound fluid MMP-9 levels from patients with diabetic foot ulcers were remarkably higher than in control subjects. This study brings to light the potential therapeutic effect that microRNAs controlling and downregulating MMP-9 levels can have in curing diabetic wounds. Similarly, studies by Gao et al. [18] and Li et al. [19] have revealed that the inhibition of MMP-9 expression encourages the healing of chronic wounds. Nguyen et al. observed that hyperbaric oxygen treatment decreases pro- and active MMP-9 in mice [20]. These studies highlight multiple methods targeting MMP-9 expression, thus altering the healing of wounds. Further studies must be conducted that focus on utilizing this information to open newer therapeutic measures for chronic non-healing wound management. Serum MMP-9 monitoring could stratify patients for such targeted therapies before irreversible tissue damage occurs.

Limitations and future directions

The small sample size of the study (N=40) precluded subgroup analyses by ulcer severity (e.g., Wagner grade) and likely obscured HbA1c correlations. The cross-sectional design also limits causal inferences, making longitudinal studies tracking MMP-9 dynamics during healing essential. Further research should include the correlation of MMP-9 with wound size, infection status, and healing trajectories in larger cohorts and aim to investigate MMP-9/TIMP-1 ratios as prognostic tools. It should also explore synergies between MMP-9 inhibitors and standard care of wounds including offloading, debridement, etc.

## Conclusions

This study demonstrates that serum MMP-9 levels are significantly elevated in individuals with chronic wounds, particularly among those with diabetes mellitus, aligning with findings from previous studies that utilized wound fluid and tissue biopsies for MMP-9 estimation. Importantly, our results show that serum-based measurement, being minimally invasive, can yield comparable insights without the procedural complexity and patient discomfort associated with collecting wound fluid or tissue samples.

Furthermore, our findings reinforce the potential utility of serum MMP-9 as a biomarker for wound chronicity. Since elevated MMP-9 levels are associated with delayed wound healing, their measurement may help clinicians assess the current state of the wound, anticipate healing trajectories, and initiate targeted therapies earlier during wound management. This has particular significance in diabetic patients, in whom early intervention can prevent complications and improve outcomes.

However, the study also highlights important limitations. Despite the expectation that serum MMP-9 levels might correlate with glycemic control, no significant association was found between MMP-9 concentrations and HbA1c levels in our diabetic cohort. This unexpected finding warrants further investigation, possibly with a larger sample size and more granular control of confounding variables. The relatively small sample size in our study may have limited the statistical power to detect such associations.

Overall, serum MMP-9 measurement offers a less invasive, clinically feasible approach to evaluating chronic wounds and tailoring management strategies. It may also serve as a future target for treatment strategies, such as those involving microRNA-based MMP-9 inhibition and hyperbaric oxygen therapy. Larger studies are needed to confirm these findings and explore their potential for everyday clinical use.
